# Effect of the Type of Herbal Preparations (Powdered Plant Material vs. Dry Ethanolic Extracts) on the Bioaccessibility of Bearberry (*Arctostaphylos uva-ursi* (L.) Spreng.) Phytochemicals in Simulated Digestion Conditions

**DOI:** 10.3390/molecules29245968

**Published:** 2024-12-18

**Authors:** Łukasz Sęczyk, Danuta Sugier, Piotr Sugier

**Affiliations:** 1 Department of Industrial and Medicinal Plants, University of Life Sciences in Lublin, 15 Akademicka Str., 20-950 Lublin, Poland; danuta.sugier@up.lublin.pl; 2 Department of Botany, Mycology and Ecology, Institute of Biological Sciences, Maria Curie-Skłodowska University, 19 Akademicka Str., 20-033 Lublin, Poland; piotr.sugier@mail.umcs.pl

**Keywords:** antioxidant activity, arbutin, artificial digestion, in vitro bioaccessibility, dry extract, ethanol extraction, herbal preparations

## Abstract

The main aim of this study was to determine the potential bioaccessibility of bearberry phytochemicals influenced by the type of herbal preparations. Herbal preparations–powdered plant materials and dry extracts obtained using various ethanol concentrations (0%, 20%, 40%, 60%, 80%, and 100%) were subjected to simulated gastric or gastrointestinal digestion for the evaluation of the bioaccessibility of the phytochemicals. The phytochemical characterization of the plant material, dry extracts, and potentially bioaccessible fractions was performed using high-performance liquid chromatography (HPLC) and spectrophotometric assays. The content of the main compounds, i.e., arbutin, hydroquinone, hyperoside, pentagalloylglucose, and picein, as well as the total phenolic content and in vitro antioxidant activity through the ABTS^•+^-scavenging activity and Fe^3+^-reducing power were determined. The bioaccessibility of arbutin, i.e., the main compound in bearberry, was high, in most cases exceeding 95%, and was generally unaffected by the experimental factors; however, the changes in the content of the other compounds, the total phenolic content, and the antioxidant activity were more prominent and influenced by the type of the herbal preparation and the stage of digestion. Given the compromise between the abundance of the bearberry phytochemicals, the antioxidant activity, and the resulting potential bioaccessibility of these phytochemicals, the dry extracts prepared with 40% ethanol seem to be the most promising for phytopharmaceutical purposes and functional food applications.

## 1. Introduction

The bearberry (*Arctostaphylos uva-ursi* (L.) Spreng.), a representative of the *Ericaceae* family, is a perennial dwarf deciduous shrub growing in forested and mountain areas of Europe, Asia, and North America [1,2,3]. The small, shiny, and coriaceous bearberry leaves are an important herbal material (*Uvae ursi folium*) with a long history of use in folk medicine since the 13th century [3,4]. Bearberry leaves have mainly been used in traditional medicine for the treatment of lower urinary system infections, but their application in heartburn, lithiasis, hydrops, and diabetes treatments are also reported [3,4,5,6]. Currently, based on their long history of use, the Committee on Herbal Medicinal Products of the European Medicines Agency officially confirmed the medicinal application of *A. uva-ursi* leaf preparations for treating mild infections in the lower urinary tract causing frequent urination and/or a burning feeling when passing urine [4]. Despite the well-known antimicrobial, anti-inflammatory, and diuretic properties strongly involved in urinary tract infection treatment, recent studies indicate a multitude of other promising biological activities of *A*. *uva-ursi* phytochemicals, e.g., antioxidant, antiproliferative, hypoglycemic, hepatoprotective, and neuroprotective activities [1,5,7,8]. Therefore, beyond phytopharmaceutical purposes, the interest in using bearberry herbal products in functional food applications is on the increase [9,10].

The most abundant phytochemical, also considered the main bioactive substance of *A*. *uva-ursi* leaves, is arbutin [1,11]. Chemically, arbutin is a hydroquinone glucoside belonging to simple phenols [9,12]. It exists in the following two isomers: natural β-arbutin (with the formal name 4-Hydroxyphenyl-β-d-glucopyranoside) and chemically or biotechnologically synthesized α-arbutin, which is commonly used in cosmetics for skin whitening [6,12,13]. The average content of β-arbutin in *A*. *uva-ursi* leaves ranges from about 5% to 15% of dry weight and depends on, e.g., the geographical origin, growing conditions, and harvest time [1,6,9]. Besides the dominant β-arbutin, its derivative methylarbutin, and its aglycone hydroquinone, the complex phytochemical composition of *A*. *uva-ursi* leaves comprises other phenolic glycosides (e.g., picein), galloylglucose derivatives (e.g., pentagalloylglucose), flavonoids (e.g., hyperoside), tannins, triterpenes, organic acids, and vitamins [1,4,5].

Due to the general preferences of consumers to use natural substances rather than synthetic analogs for the prevention or treatment of some medicinal conditions, a growing trend of using herbs and herbal products is observed [4,12]. The administration of complex mixtures of phytochemicals can provide additive or synergistic effects and enhance the advantageous pro-health properties of bioactive components [14].

Although many studies, especially in vitro and in cell lines, demonstrate the strong biological activity of natural plant compounds, including arbutin and other *A*. *uva-ursi* phytochemicals, their in vivo efficacy may be limited [7,11,15]. Low bioaccessibility and, consequently, low bioavailability are regarded as one of the determinants of their efficacy; therefore, various strategies have been developed to overcome this potentially unfavorable effect, such as the optimization of the form of administration, supply, dosage, formulation, and routes of delivery [16,17].

The most popular forms of the oral administration of herbal preparations are infusions, tinctures, dry extracts, and isolates. In addition, herbal powders are often used in dietary supplement production and for food fortification purposes [18]. On the one hand, the administration of powdered raw herbal plant material provides the organism with all of the phytochemical constituents contained therein; on the other hand, the presence of the endogenous plant matrix can negatively affect the extractability of bioactive compounds during digestion, thereby limiting their potential beneficial health effects [17,19]. The application of concentrated dry extracts can help in the easier achievement of an appropriately high level of phytochemicals to exert biological functions; however, during the preparation and further processing of extracts, the potential loss in bioactive constituents caused by the solvent-dependent partial extractability and possible degradation of compounds may occur [19,20].

Given the above considerations, the main objectives of this study were to characterize the *A*. *uva-ursi* phytochemical profile, in vitro antioxidant activity, extractability, and stability during the preparation of dry extracts, depending on the various ethanol concentrations, and then to determine the in vitro bioaccessibility of phytochemicals from the tested herbal preparations (powdered plant material and dry extracts) at the gastric and intestinal stages of simulated digestion.

## 2. Results

### 2.1. Phytochemical Characterization of Herbal Preparations

The content of the main individual phytochemicals in the bearberry herbal preparations and the stability of compounds during extract drying are presented in Table 1.

The abundance of the analyzed compounds in the herbal preparations was as follows: arbutin > hydroquinone > hyperoside > pentagalloylglucose > methylarbutin > picein. The results showed that the concentration of the phytochemicals and their stability during extract drying depended on the solvent variant. The contents of individual phytochemicals determined in the powdered plant material (PPM) reflect the extractability of the analyzed substances in the applied solvent variants. The highest extractability of arbutin (86.69 mg/g) and picein (2.04 mg/g) was observed in the process variant, whereas hydroquinone (18.17 mg/g), hyperoside (10.37 mg/g), and methylarbutin (3.68 mg/g) exhibited the highest extractability in the 20E and pentagalloylglucose (6.59 mg/g) in the 40E. Due to the different levels of extractability of the phytochemicals influenced by the solvent variants, the highest amounts determined in the different solvent variants (mentioned above) were considered as those reflecting the content of the compound in the powdered plant material. The preparation of dry extracts (DEs) yielded a more concentrated form of the phytochemicals. The highest concentrations of arbutin, hyperoside, and picein were observed in the W, i.e., 199.11 mg/g, 22.71 mg/g, and 4.20 mg/g, respectively. The highest levels of hydroquinone and methylarbutin, i.e., 29.58 mg/g and 6.34 mg/g, respectively, were detected in the 20E, and the highest concentration of pentagalloylglucose, i.e., 12.02 mg/g, was found in the 40E. The analyzed compounds were relatively stable during the dry extract preparation, especially in the case of the 20E–E, where the stability was higher than 95% in most cases. The lowest stability for all of the compounds during the dry extract preparation was observed in the W. The most stable compound was hyperoside, with a stability close to 100% in the 20E–E.

The total phenolic content and antioxidant activity of the bearberry herbal preparations evaluated based on their ability to scavenge ABTS^•+^ and reduce Fe^3+^, and the stability of the phytochemicals during the extract drying, are shown in Table 2.

The highest extractability of the phytochemicals from the powdered plant material reflected by the total phenolic content was determined in the 60E (261.31 mg GAE/g), while the application of the 40E resulted in the highest ABTS^•+^ scavenging activity (834.97 mg TE/g) and Fe^3+^-reducing power (326.15 mg TE/g). However, in each case, there was no statistically significant difference between the ethanol concentrations in the range from 20% to 80% (20E–80E). The application of the 40E for the preparation of dry extracts resulted in the highest abundance of total polyphenols (480.12 mg GAE/g), ABTS^•+^ scavenging activity (1544.36 mg TE/g), and Fe^3+^-reducing power (318.56 mg TE/g). In both the PPM and DE, the lowest total phenolic content and antioxidant activities were determined after the water extraction procedure. In addition, despite the lack of statistically significant differences between the applied solvents, the water extraction procedure caused the lowest stability of phytochemicals during extract drying, whereas the relatively high stability was maintained in the 20E–E, which was >90% for all of the measured parameters.

### 2.2. In Vitro Bioaccessibility Determination

**Figure 1:**
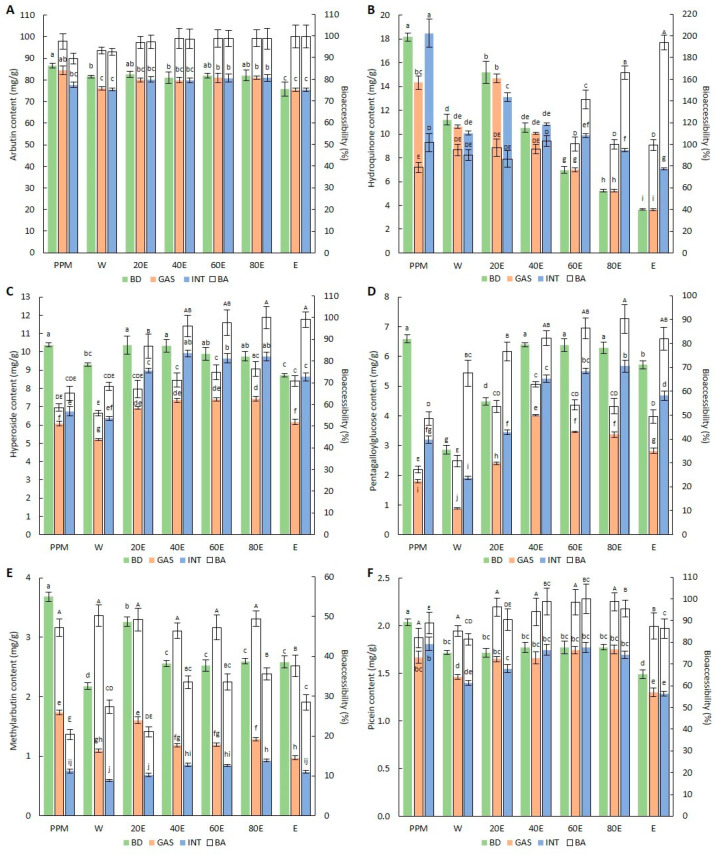
Effect of simulated digestion on the potentially bioaccessible content and the bioaccessibility percentage of arbutin (**A**), hydroquinone (**B**), hyperoside (**C**), pentagalloylglucose (**D**), methylarbutin (**E**), and picein (**F**). Bars represent means (*n* = 3) ± SD. Means followed by different superscript lowercase letters for quantitative results and superscript uppercase letters for the bioaccessibility percentage differ significantly. PPM—powdered plant material, W—water extract, 20E—20% ethanol extract, 40E—40% ethanol extract, 60E—60% ethanol extract, 80E—80% ethanol extract, and E—100% ethanol extract; BD—sample before digestion, GAS—sample after gastric digestion, INT—sample after gastrointestinal digestion, and BA—bioaccessibility percentage.

The effect of the simulated digestion on the content of potentially bioaccessible compounds and their bioaccessibility percentage from the applied herbal preparations are shown in Figure 1.

The two-way ANOVA analysis showed a statistically significant impact (*p* ≤ 0.05) of the type of digested material, the stage of digestion, and their interaction on the content of each compound. The two-way ANOVA analysis of the bioaccessibility percentage showed a statistically significant impact (*p* ≤ 0.05) of the type of digested material, the stage of digestion, and their interaction in the case of hydroquinone, hyperoside, pentagalloylglucose, and methylarbutin. In turn, a statistically significant impact (*p* ≤ 0.05) of only the type of digested material was observed in the case of arbutin and picein.

Considering arbutin (Figure 1A), the simulated gastric and gastrointestinal digestion had only a slight (in most cases, statistically insignificant) negative effect on its bioaccessible content and the corresponding bioaccessibility percentage, which was the most noticeable in the dry extracts (20E–E). The application of the 20E–E, regardless of the digestion stage, resulted in a high percentage of bioaccessibility (>97%). Statistically lower bioaccessible arbutin content, compared to that in the undigested material, was found in the W both after the gastric and gastrointestinal digestion, and in the powdered plant material after the gastrointestinal digestion, where the bioaccessibility percentages were 93.2%, 92.7%, and 89.6%, respectively.

The phenomena observed after the digestion were much more diversified in the case of hydroquinone (Figure 1B). The gastric digestion of all of the dry extracts (W-E) had no significant influence on the hydroquinone content and corresponding bioaccessibility, which ranged from 94.9% (W) to 100.4% (60E). Nevertheless, the application of the powdered plant material for the gastric digestion resulted in the lowest percentage of bioaccessibility of this compound (78.8%) compared to the other herbal preparations. In turn, no significant influence was observed after the gastrointestinal digestion. Interestingly, the content and percentage of the bioaccessibility of hydroquinone after the gastrointestinal digestion were strongly differentiated by the extract type. The application of the W and 20E for the gastrointestinal digestion resulted in a relatively slight reduction in the bioaccessible hydroquinone content (with a bioaccessibility of 90.1% and 86.5%, respectively); however, the results obtained in the extracts prepared with the higher concentrations of ethanol, especially the 60E–E, showed a statistically significant increase in the content of this substance in comparison with the dry extract before the digestion step, and in the bioaccessibility percentage, which was significantly higher than 100%, i.e., 141.6%, 165.9%, and 193.9% in the 60E, 80E, and E, respectively.

The hyperoside content (Figure 1C) was mainly negatively affected after the gastric digestion. The statistically significant reduction in its content was the most notable in the powdered plant material (with a bioaccessibility of 58.6%) and W (with a bioaccessibility of 55.9%). In the other samples (20E–E), it ranged from 67.1% (20E) to 76.3% (80E). All of the herbal preparations analyzed after the gastrointestinal digestion process were characterized by higher bioaccessibilities than after the gastric digestion. The highest bioaccessibility percentages in the gastrointestinal digestion stage were found in the case of the dry extracts prepared with the higher concentrations of ethanol (40E–E), where they ranged from 96.2% (40E) to 100.0% (80E), whereas the most significant reduction in the hyperoside content compared to the undigested material was found in the PPM (with a bioaccessibility of 65.1%).

The simulated digestion strongly decreased the content of pentagalloylglucose (Figure 1D), especially after the gastric digestion. It was found that the application of the PPM resulted in the lowest bioaccessibility percentage among all of the herbal preparations, i.e., 27.2% and 48.5% after the gastric and gastrointestinal digestion steps, respectively, whereas the highest bioaccessibilities were observed in the 40E (62.9%) (without statistically significant differences between variants 20E and 80E) and 80E (90.2%) (without statistically significant differences between variants 40E and E) after the gastric and gastrointestinal digestion, respectively.

The methylarbutin content (Figure 1E) was strongly reduced after both the gastric and gastrointestinal digestion. However, in contrast to pentagalloylglucose, significantly lower bioaccessibilities were observed after the intestinal stage of digestion. After the gastric digestion, the lowest percentage of bioaccessibility was determined in the E (37.6%) and the highest percentage was exhibited by the W (50.2%), but without statistically significant differences from the other herbal preparations. After the gastrointestinal digestion, the lowest bioaccessibility was observed in the PPM (20.4%) (without a statistically significant difference from the 20E), while the highest level (35.6%) was calculated in the 80E (without a statistically significant difference between the 40E and 60E).

In the case of picein (Figure 1F), there was no statistically significant difference in the content and bioaccessibility percentage between the stages of simulated digestion. After the gastric and gastrointestinal digestion, a significant decrease in its content, in comparison with the undigested herbal preparation, was observed in the PPM, W, and E. The lowest percentage of bioaccessibility was found in the powdered plant material (PPM—82.0%) after the gastric digestion and in the W (81.4%) after the gastrointestinal digestion. The highest bioaccessibility percentage was observed in the 80E (98.8%) after the gastric digestion (without statistically significant differences from the other extracts) and in the 60E (100.1%) after the gastrointestinal digestion (without statistically significant differences from the powdered plant material and the 20E–E).

**Figure 2:**
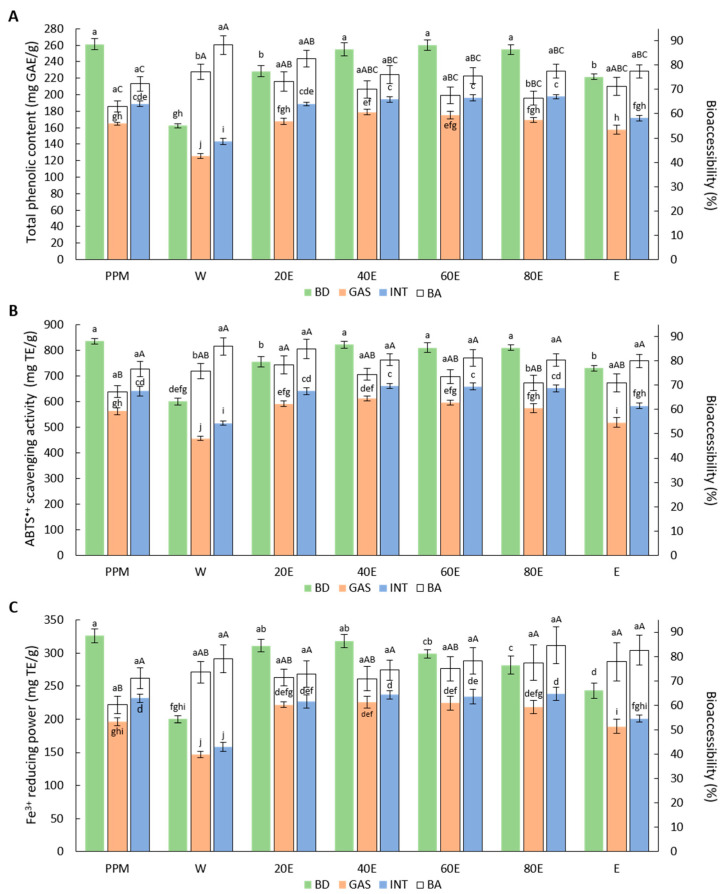
Effect of simulated digestion on the potentially bioaccessible total phenolic content (**A**), ABTS^•+^ scavenging activity (**B**), and Fe^3+^-reducing power (**C**). Bars represent means (*n* = 3) ± SD. Means followed by different superscript lowercase letters differ significantly for quantitative results. For the bioaccessibility percentage, means followed by different superscript lowercase letters within the same type of digested material indicate a statistically significant difference between the stage of digestion and the means, followed by different superscript lowercase letters and uppercase letters within the same stage of digestion, which indicate a statistically significant difference between the types of digested material. PPM—powdered plant material, W—water extract, 20E—20% ethanol extract, 40E—40% ethanol extract, 60E—60% ethanol extract, 80E—80% ethanol extract, and E—100% ethanol extract; BD—sample before digestion, GAS—sample after gastric digestion, INT—sample after gastrointestinal digestion, and BA—bioaccessibility percentage.

The influence of the simulated digestion on the total phenolic content and antioxidant activities is shown in Figure 2.

The two-way ANOVA analysis showed a statistically significant impact (*p* ≤ 0.05) of the type of digested material, the stage of digestion, and their interaction on the total phenolic content, ABTS^•+^ scavenging activity, and Fe^3+^-reducing power (expressed quantitatively). For each measured parameter, a two-way ANOVA analysis of the results of the bioaccessibility percentage showed a statistically significant (*p* ≤ 0.05) impact of the type of digested material and the stage of digestion, without a significant effect of their interaction. The analysis of the total phenolic content (Figure 2A) indicated a statistically significant decrease in the concentration of potentially bioaccessible phytochemicals, in particular after the gastric digestion procedure. Considering the type of herbal preparation, the lowest percentage of bioaccessibilities was observed in the powdered plant material, i.e., 63.1% after the gastric digestion and 72.3% after the gastrointestinal digestion. Unexpectedly, despite the low abundance of phytochemicals before the digestion, the highest percentage of bioaccessibility after the gastric and gastrointestinal digestion was noticed in the W, i.e., 77.3% and 88.3%, respectively (without a statistically significant difference from the 20E).

Similarly to the total phenolic content, the ABTS^•+^ scavenging activity (Figure 2B) significantly decreased after the digestion, and this effect was particularly noticeable after the gastric digestion process. In addition, the lowest percentage of bioaccessibilities among the tested herbal preparations was also obtained in the powdered plant material, i.e., 67.3% after the gastric digestion and 76.6% after the gastrointestinal digestion. The highest bioaccessibility was obtained in the 20E after the gastric digestion and in the W after the gastrointestinal digestion; however, in both cases, there were no statistically significant differences from the other dry extracts.

The results of the Fe^3+^-reducing power (Figure 2C) indicate a significant decrease in this activity after the digestion but without significant differences between the stages of digestion in most cases. The lowest percentage of bioaccessibility was found in the powdered plant material, i.e., 60.2% and 71.0% after the gastric and intestinal stages of digestion, respectively. The highest bioaccessibility was determined in the E (78.3%) after the gastric digestion and in the 80E (85.9%) after the gastrointestinal digestion, but without a statistically significant difference from the other dry extracts.

## 3. Discussion

The profile and content of bioactive phytochemicals are commonly considered as the primary distinguishing features of the quality of medicinal herbs and herbal preparations.

Taking into consideration the possible different degrees of extractability of phytochemicals influenced by the various physicochemical characteristics of individual compounds and their affinity for the solvent composition, it was assumed in this study that the maximal content of substances or antioxidant activity determined in the tested extracts (initial liquid extracts) corresponded with those in the plant material (Table 1 and Table 2).

In this study, the content of arbutin, i.e., the primary and marker compound in *A. uva-ursi*, assumed to correspond with that in the plant material, was approximately 8.7% (86.69 mg/g (W)) (Table 1). A relatively similar concentration of arbutin in bearberry leaves was also found in other studies, i.e., 8.2% [21], 8.5% [22], and 9.5% [23]. Furthermore, such content is within the range previously reported for Polish populations of bearberry, from 5.6% to 9.7% [24], and western Balkan populations, from 7.0% to 9.4% [3], but slightly lower than the range reported for Spanish populations, from 8.7% to 21.1% [25]. Nevertheless, the content of arbutin (i.e., ≥7%) determined in the present study meets the formal requirements of the European Pharmacopoeia, classifying such plant material as herbal material to be officially used for medicinal purposes [3,24].

The content of the other individual compounds corresponding with that in the plant material (Table 1) was also within the range reported previously for Polish populations [24]; however, some differences were found in comparison with other reports. For example, in our study, a significantly higher content of hyperoside (10.37 mg/g (20E) vs. 2.92 mg/g), and a significantly lower content of pentagalloylglucose (6.59 mg/g (60E) vs. 21.89 mg/g) and picein (2.04 mg/g (W) vs. 12.32 mg/g), was found in comparison with the results reported by Olennikov and Chekhirova [21]. Similarly, in the case of the total phenolic content, ABTS^•+^ scavenging activity, and Fe^3+^-reducing power, the present results were within the range determined in Polish populations [24]. Nonetheless, the total phenolic content was higher than that reported for Spanish populations, i.e., 261.31 mg GAE/g (60E) vs. 103–206 mg GAE/g, whereas the antioxidant properties were not investigated [25].

The phytochemical composition of herbs depends on a multitude of factors. The genetic diversity, geographical origin, habitat, environmental conditions (e.g., weather and soil conditions), and growing stage are commonly reported as factors influencing the natural variability of phytochemicals in *A. uva-ursi*. Furthermore, the differences in the content of phytochemicals in bearberry plant material assessed in various studies can be caused by the different methods of preservation and post-harvest treatment of raw materials, further processing and storage, and different methodological approaches, including extraction techniques and applied analytical procedures [1,3,4,25,26].

So far, the effect of the solvent composition, in particular the use of water and ethanol solutions, on the extractability of *A. uva-ursi* compounds, their stability during dry extract preparation, and their concentration in dry extracts, is rather scarcely described. The effect of water and 70% ethanol extraction on the phytochemical composition and antioxidant activity in an *A. uva-ursi* population was investigated in a study conducted by Sugier et al. [26]. The authors reported similar trends to those determined in the present study (Table 1 and Table 2), i.e., the comparable extractability of arbutin in water and ethanol solutions, the higher extractability of hydroquinone in water, the higher extractability of hyperoside in an ethanol solution, and the higher total phenolic content, scavenging activity against ABTS^•+^, and Fe^3+^-reducing power. In the case of methylarbutin, pentagalloylglucose, and picein, the effect of the solvent was more diversified and dependent on the analyzed population [26]. Shamilov et al. investigated the effect of water, 40% ethanol, 70% ethanol, and 95% ethanol extraction on the antioxidant activity of bearberry extracts [27]. Similarly, stronger antioxidant activity (antiradical activity against superoxide, DPPH, nitrosyl, and hydroxyl radicals) was observed in the 40% and 70% ethanol variants compared to the other extracts (water and 95% ethanol); however, the results cannot be quantitatively compared because of their different expression (IC_50_) [27].

The basic phytochemical characterization of *A. uva-ursi* dry extracts was performed by Azman et al. [9] and Dell’Annunziata et al. [28]. In comparison to the present study, where the total phenolic content ranged from 162.34 to 259.78 mg GAE/g in the W and 60 E, respectively (Table 2), a lower content of polyphenols (i.e., 102.11 mg GAE/g) was obtained by Azman et al. in 50% dry ethanol extracts [9], whereas a higher content (i.e., 345 mg GAE/g) was determined by Dell’Annunziata et al. in commercial dry extracts (solvent unspecified) [28]. The content of individual compounds and the stability of the phytochemicals were not monitored in the cited studies. Interestingly, we found the very high antioxidant potential of the *A. uva-ursi* dry extracts as reflected by the ABTS^•+^ scavenging activity, where 1 g of dry extract was approximately 50% more effective than the Trolox reference substance (depending on the extract type, 1 g of dry extract corresponds to from 1.44 g to 1.54 g Trolox equivalents) (Table 2), which predisposes the extracts to be used as a source of natural antioxidants, e.g., in food systems.

Besides the aforementioned initial concentrations of compounds in plant materials and the different analytical procedures employed, differences in the phytochemical profile of herbal preparations between studies can be caused by a multitude of other factors.

We found that the majority of the analyzed compounds, i.e., arbutin, hydroquinone, hyperoside, methylarbutin, and picein, were the most extractable in the more polar environments, i.e., water and 20% ethanol, which is consistent with their polar character, related to their molecular structure and physicochemical properties, ensuring good solubility in such solvents [29]. Nonetheless, besides the physicochemical nature of the analyzed compounds and extractant properties (e.g., polarity), the extractability of plant substances is also a result of other factors, including herbal material properties, like the plant matrix micro- and macrostructure and the chemical composition, the extraction technique (conventional or accelerated), and the conditions (e.g., temperature and time) influencing the release of compounds during extraction [4,19,30].

Moreover, the procedures applied during the further processing of extracts, e.g., drying, can change the phytochemical composition of the final herbal products. In particular, phenolic compounds as well-known antioxidants are susceptible to oxidative degradation during processing (they protect other molecules against oxidation with simultaneous self-oxidization). External conditions, like high temperatures or the presence of oxygen and water, can promote phytochemical degradation and loss in dry herbal extracts [20,31,32].

Our study showed the relatively high stability of the individual bearberry phytochemicals, the total phenolic content, and the antioxidant activities, which were mostly higher than 95% or even close to 100%, particularly in the case of the extracts prepared with moderate ethanol concentrations (40% and 60%) (Table 1 and Table 2). Nonetheless, the lowest stability during drying was observed for the water extracts, where the highest loss in the phytochemicals occurred despite the application of lyophilization during the entire drying process, which is recognized as a very mild technique in terms of the temperature of the drying. Therefore, it may be speculated that the temperature condition was a less relevant determinant of compound stability than the extractant used. Predictably, the high stability of the ethanolic extracts, especially those prepared with the moderate concentrations of ethanol, was attributed to the high abundance of phytochemicals other than those identified (Table 1), as reflected by the high total phenolic content and the strong antioxidant activity (Table 2), thus ensuring self-protecting effects during drying. As in the case of the extractability, the stability of the phytochemicals can also be influenced by their concentration and individual physicochemical character (susceptibility to oxidation), and the surrounding environment (e.g., the medium/solvent, temperature, and occurrence of other molecules) [20,33,34].

Although the promising phytochemical profile determined after solvent extraction is well known, increasing numbers of recent studies show that the in vivo beneficial properties and pharmacological relevance of herbal products can be restricted by the low bioaccessibility of bioactive substances. Therefore, bioaccessibility determination studies have been carried out to better predict the biological potential of *A. uva-ursi* [19,35,36,37].

Bioaccessibility is a result of such key factors as the release of phytochemicals from the matrix, stability in the digestion environment, and, subsequently, the occurrence in an unconjugated, absorbable form [38,39,40,41]. During digestion, such physicochemical factors as the temperature, the presence of oxygen, especially at the initial stage of digestion (during meal mincing at the oral phase), pH changes, and interactions with electrolyte constituents can promote the conversion of polyphenols to by-products and their degradation [39,42,43]. Furthermore, it is known that phenolic substances can interact with other plant matrix components, e.g., proteins, lipids, and carbohydrates, as well as biochemical constituents of digestive fluids, such as enzymes and bile salts, thus decreasing the level of free–bioaccessible compounds [38,44,45,46]. The loss in the phytochemical content or activity caused by the aforementioned factors, in comparison with the initial material, results in a bioaccessibility that is lower than 100%.

On the one hand, the expression of bioaccessibility as a percentage share of bioaccessible compounds in relation to their abundance in the material before digestion allows for the estimation of the behavior of compounds (release and stability) in digestion conditions, depending on the applied dose (the relative value), i.e., the higher the bioaccessibility percentage, the higher the resistance of the compound to the digestion environment. On the other hand, quantitative determinations of phytochemical content/activity in potentially bioaccessible fractions help to determine the specific load of the analyzed substances after the digestion of a certain dose of the herbal preparation. For example, despite a high percentage of bioaccessibility of an analyzed compound, its low initial abundance (or dose) may result in a lower specific concentration compared to that of other compounds. In turn, despite its lower percentage of bioaccessibility, a high abundance of the compound in the initial material (or dose) can compensate for the negative influence of the digestion environment. Therefore, the content of potentially bioaccessible phytochemicals and the bioaccessibility percentage were determined in this study.

The analysis of the potential bioaccessibility of arbutin showed that it was relatively high (>90%) and, in general, unaffected or slightly affected by the herbal preparation type and the digestion stage, which is a promising finding in terms of using bearberry herbal products as a natural source of bioaccessible arbutin; however, it should be noted that the pharmacological activity of complex herbal preparations is often a result of a combination of the activity of many compounds (e.g., the occurrence of synergistic or cumulative effects) [14]. Therefore, the bioaccessibility of other less abundant compounds that could be important for the phytopharmaceutical potential of *A. uva-ursi* herbal preparations was also evaluated.

It was found that the effect of the analyzed experimental factors was more complex in the case of other compounds, especially hydroquinone, where the bioaccessibility strongly varied depending on the type of digested material and the stage of digestion. Unexpectedly, the hydroquinone bioaccessibility highly exceeded 100% at the intestinal stage of digestion of the 60E–E, despite the initial low concentration of the compound (Figure 1B), which indicates its formation during intestinal digestion. A study conducted by Braga et al. reported that, besides its natural occurrence in plant material, hydroquinone can be formed by the degradation of arbutin [6]. However, the accelerated degradation conditions applied in the cited study (0.1 M NaOH alkaline hydrolysis, 1 M HCl acidic hydrolysis, oxidation with 0.3% H_2_O_2_, and thermal treatment up to 105 °C) were much more extreme than those occurring during in vitro digestion. Considering the high stability of arbutin, reflected by its high bioaccessibility in the digestion conditions (Figure 1A), it can be speculated that hydroquinone could be formed from other possibly less stable hydroquinone derivatives like methylarbutin (where its content was significantly reduced after the digestion—Figure 1E) and/or other unidentified (due to the lack of commercially available standards for the UV–Vis HPLC analysis) hydroquinone-containing compounds, like arbutin galloyl derivatives reported in other studies [22,47], after mass spectrometry analysis, which presumably could be characterized by the better extractability at higher concentrations of ethanol. Given the discussion concerning the potential toxicological risk of free hydroquinone [2,6], its high bioaccessibility should be considered during the assessment of the biological potential of *A. uva-ursi* herbal preparations, and studies are needed to gain insight into the factors influencing the possible hydroquinone formation during digestion.

Furthermore, the effect of such experimental factors as the digestion stage and the type of digested material was pronounced in the case of hyperoside and pentagalloylglucose. In both cases, the presence of the solid-state plant matrix during the digestion process (digestion of powdered plant material) resulted in a significant decrease in the bioaccessibility, compared to the corresponding amounts of dry extracts, with a comparable concentration of compounds, in particular in the extracts prepared at ethanol concentrations ≥40% (Figure 1C,D). Therefore, this effect can be attributed to the interaction of these compounds with the plant matrix, resulting in a decrease in their free level. In addition, the analysis of the bioaccessibility of these compounds from dry extracts (where the plant matrix effects seem marginal) may indicate reversible interactions with biochemical components of digestive fluids (enzymes), reflected by the higher bioaccessibility of the phytochemicals after the intestinal than the gastric digestion procedure (Figure 1C,D). It has been reported that the affinity of phenolic substances to macromolecules, like digestive enzymes, is strongly dependent on the pH value [48,49]. Probably, the low pH value at the gastric stage of digestion resulted in the more efficient binding of hyperoside and pentagalloylglucose in comparison with the neutral pH conditions present at the intestinal stage of digestion, which resulted in the release of the compounds and the higher bioaccessibility at the subsequent step of digestion (Figure 1C,D). Nonetheless, the influence of other digestion fluid components or changes in the solubility of the phytochemicals induced by the digestion environment cannot be ruled out. Opposite effects, i.e., significantly higher bioaccessibility at the gastric than the intestinal stage of digestion, were observed for methylarbutin (Figure 1E), whereas the picein bioaccessibilities were comparable (Figure 1F). The differentiation in bioaccessibilities among the compounds indicates their specific interactions with the digestion environment, depending on their chemical type and individual properties.

Despite the plant matrix and digestion-related effects, it was found that the bioaccessibility of individual compounds was dependent on the variant of the applied dry extract. In most cases, the highest bioaccessibilities of individual compounds were determined in the 40E–80E, but the lowest values of the parameter were exhibited by the W (Figure 1). The bioaccessibility of individual compounds, in general, was consistent with the abundance of phytochemicals, reflected by the total phenolic content and antioxidant activity (Table 2). This observation also suggests the significant contribution of other (unidentified) compounds to the response of individual compounds to the digestion conditions. Interestingly, the higher total phenolic content and antioxidant activity coincided with the higher stability during drying and the higher bioaccessibility of the ethanol extracts, which indicates their promising profile in terms of the resistance of the compounds to external conditions.

Unexpectedly, the analysis of the total phenolic content and antioxidant properties after the digestion of the water extracts showed one of the highest bioaccessibility percentages among the extracts (Figure 2). Presumably, this effect may have resulted from the high bioaccessibilities of other unidentified compounds contained in the extracts and/or the higher contribution of more stable compounds in the extract profile caused by the degradation of less stable compounds during the dry extract preparation. It should be noticed that, despite the high percentage of bioaccessibility, the specific concentration and activity of potentially bioaccessible compounds (expressed quantitatively in mg/g) in the water dry extract was low (Figure 2).

## 4. Materials and Methods

### 4.1. Reagents and Chemicals

The extraction solvent, i.e., absolute ethanol (≥99.8%), was purchased from Supelco (Bellefonte, PA, USA). Digestion fluid components, i.e., α-amylase (from hog pancreas), pepsin (from porcine gastric mucosa), pancreatin (from porcine pancreas), and bile extract (bovine); standards for chromatographic determinations, i.e., β-arbutin (≥98%), hydroquinone (≥99%), hyperoside (≥95%), pentagalloylglucose (≥96%), methylarbutin (≥97%), and picein (≥98%); HPLC-grade eluent components, i.e., acetonitrile and formic acid; standards and chemicals for spectrophotometric determinations (the total phenolic content and antioxidant activity), i.e., gallic acid (≥98%), Folin–Ciocalteu’s reagent, Trolox (≥97%), 2,2′-azino-bis 3-ethylbenzothiazoline−6-sulfonic acid (ABTS), and 2,4,6-Tris(2-pyridyl)-s-triazine (TPTZ) were supplied by Sigma-Aldrich (St. Louis, MO, USA). All other reagents were of analytical grade.

### 4.2. Plant Material

The *Arctostaphylos uva-ursi* species used in the experiment was identified by Mykhaylo Chernetskyy, a taxonomist from the Botanic Garden of Maria Curie-Skłodowska University in Lublin, based on the reference material no. 4406P from the collection of the Botanical Garden of Maria Curie-Skłodowska University in Lublin. Bearberry leaves were harvested from plants growing in pine forests located in the area of Krzywda (Lublin Voivodeship) in eastern Poland. Fresh leaves were dried in a laboratory-forced convection drying chamber at 40 °C for two days. The dried leaves were finely ground using an electric grinder and sieved (passing through a 60-mesh nylon sieve) to obtain powdered plant material for further analysis.

### 4.3. Extraction Procedure and Dry Extract Preparation

Phytochemicals were extracted from powdered plant material using water and various concentrations of absolute ethanol, i.e., 20, 40, 60, 80, and 100% (*v*/*v*). The samples were labeled as W, 20E, 40E, 60E, 80E, and E, respectively. The powdered plant material was subjected to a three-step ultrasound-assisted extraction procedure in a water bath. In the first step, the material (1.5 g) was poured into 25 mL of the solvent and incubated at 45 °C for 100 min with manual shaking every 10 min for 5 s. The samples were centrifuged (5000 RPM, 10 min, and 20 °C) and the supernatants were separated. Then, insoluble particles were subjected to another two steps. The residues of each plant material were poured with 5 mL of the solvent, incubated for 10 min in an ultrasonic water bath set at 45 °C, and centrifuged (5000 RPM, 10 min, and 20 °C). Supernatants collected from each step were combined and, using external pressure, passed through a plastic Buchner funnel equipped with cellulose filter paper.

The obtained liquid extracts were used for the analysis of the extractability of the phytochemicals influenced by the solvent variants and their corresponding initial concentration (or antioxidant activity) for the determination of the stability of the compounds during the extract drying process.

An analogical procedure was applied using 10-fold greater amounts of powdered plant material and a correspondingly higher amount of the solvent to obtain initial liquid extracts for the preparation of dry extracts. To obtain the dry extracts, the liquid extracts W and 20E were freeze-dried, the 40E and 60E were partially evaporated under reduced pressure using a rotary evaporator and then freeze-dried, and the 80E and E were fully evaporated in a rotary evaporator. After drying, the amount of extracts was quantified using a laboratory scale, and the dry extract yield and the plant material/dry extract ratio were calculated (Supplementary Table S1). Raw dry extracts were manually pulverized using a mortar and pestle into homogenous powder.

Part of the powdered dry extracts was re-dissolved in corresponding solvents (5 min, 45 °C, and an ultrasound water bath) for the determination of their phytochemical concentration and antioxidant activity, as well as the stability of the compounds during the dry extract drying process.

### 4.4. Experiment Design and Artificial Digestion Procedure

To compare the effect of the form of administration (powdered plant material vs. dry extract) and, consequently, the endogenous plant matrix (whole plant matrix vs. extractable compounds) on the in vitro bioaccessibility of bearberry phytochemicals, excluding the effect of their concentration during the preparation of dry extracts, powdered plant material (PPM) and dry extracts (DEs) were used in the digestion experiment at corresponding doses (i.e., DEs in amounts obtained with a given amount of PPM regarding the dry extract yield). The artificial digestion was performed according to the INFOGEST procedure [50] with the following modifications. Since the general recommendations of the protocol are intended for commonly consumed fresh and, therefore, well-hydrated food products, as well as the assumed natural lower intake of herbal products, especially in the dehydrated form, the amount of material subjected to digestion was downgraded 10 times, i.e., instead of 5 g of the “typical” meal recommended by the standard procedure, 0.5 g of PPM or a corresponding amount of DE was applied [35]. The crucial digestion-related experimental conditions, i.e., the electrolytic composition of the digestion fluids, enzymes, bile salt concentrations, pH, temperature, and the duration of the digestion phase, were strictly implemented from the standard protocol [50]. Powdered plant material and dry extracts were subjected to successive oral and gastric digestion to determine the bioaccessibility of the phytochemicals after the gastric stage of digestion or to oral, gastric, and intestinal digestion to determine their bioaccessibility after gastrointestinal digestion. After a simulated digestion process, potentially bioaccessible fractions were obtained by centrifugation (9000 RPM, 10 min, and 4 °C), followed by the ultrafiltration of the supernatants using a centrifugal filter unit with a 10 kDa molecular weight cut-off membrane [35].

### 4.5. Phytochemical Analysis

#### 4.5.1. Individual Compounds—HPLC Analysis

The separation and quantification of the phytochemicals were performed using a Varian ProStar HPLC system coupled with a UV–Vis detector (Varian Inc., Walnut Creek, CA, USA). The samples were separated on an octadecylsilane (C18) reversed-phase Gemini column (5 μm, 110 Å, and 250 mm × 4.6 mm) (Phenomenex, Torrance, CA, USA) using 0.1% HCOOH in H_2_O (*v*/*v*) (A) and 0.1% HCOOH in ACN (*v*/*v*) (B) eluents at the following gradient modes: pre-run—96% (A), 5 min—96% (A), 30 min—78% (A), 45 min—75% (A), 50 min—0% (A), 55 min—0% (A), 60 min—96% (A), and 65 min—96% (A) at a constant flow rate of 1.0 mL/min and a temperature set at 25 °C. The chromatograms were registered at 280 nm for arbutin, hydroquinone, pentagalloylglucose, methylarbutin, and picein, and at 350 nm for hyperoside. The phytochemicals were identified by comparing their retention times with that of a reference standard (Supplementary Figure S1) and quantified using an external standard curve (Supplementary Table S2). Examples of chromatograms of bearberry phytochemicals are presented in Supplementary Figures S2–S7.

#### 4.5.2. Total Phenolic Content and Antioxidant Activity

Spectrophotometric assays of the total phenolic content, antiradical activity against ABTS^•+^, and Fe^3+^-reducing power were performed according to the methods developed by Singleton and Rossi [51], Re et al. [52], and Benzie and Strain [53], respectively, with previously described adaptation to microplate reader measurements [24].

### 4.6. Expression of Results and Calculations

The results of the individual compound determinations were expressed as mg of compound per g dry weight of powdered plant material (PPM), dry extract (DE), or powdered plant material equivalent for dry extracts (DE_PPMeq_). The results of the total phenolic content and antioxidant activities were expressed as mg of gallic acid equivalents (GAEs) or Trolox equivalents (TEs) per g of PPM, DEs, or DE_PPMeq_, respectively. In DE_PPMeq_, the concentration of compounds or antioxidant activity was expressed per amount of dry extract corresponding to (the obtained from) g of PPM (calculated based on the dry extract yield—Table S1). DE_PPMeq_ was used for the calculations of the stability and potential bioaccessibility percentage of the phytochemicals. The expression of the results as DE_PPMeq_ allowed for a comparison of the results for the powdered plant material and the dry extracts used at corresponding doses.

#### 4.6.1. Phytochemical Stability During Extract Drying

The stability of the phytochemicals during the preparation of the dry extracts was calculated using the following equation:

(1) $$Stability\left(\%\right)=\frac{{PC\left(A\right)DE}_{PPMeq}}{IPC\left(A\right)PPM}\times 100$$

where PC(A) DE_PPMeq_—the phytochemical content (PC) or antioxidant activity (A) of the dry extracts, expressed as the powdered plant material equivalent (DE_PPMeq_); IPC(A) PPM—the initial phytochemical content (IPC) or antioxidant activity (A) of the powdered plant material (PPM), determined based on the analysis of liquid extracts (before drying).

#### 4.6.2. Bioaccessibility Percentage

The bioaccessibility percentage of the powdered plant material and dry extracts was calculated as follows:

(2) $$Bioaccessibility\left(\%\right)=\frac{BPC\left(A\right)PPM({DE}_{PPMeq})}{IPC\left(A\right)PPM({DE}_{PPMeq})}\times 100$$

where BPC(A) PPM(DE_PPMeq_)—the bioaccessible phytochemical content (BPC) or antioxidant activity (A) of the powdered plant material (PPM) or dry extracts, expressed as the powdered plant material equivalent (DE_PPMeq_); IPC(A) PPM(DE_PPMeq_)—the initial (before digestion) phytochemical content (IPC) or antioxidant activity (A) of the powdered plant material (PPM) or dry extracts, expressed as the powdered plant material equivalent (DE_PPMeq_).

### 4.7. Statistical Analysis

The results represent the means of three experimental repetitions ± standard deviation. The statistical analysis was carried out using Statistica 6.0 software (Stat. Soft, Inc., Krakow, Poland). For the determination of statistically significant differences between the studied experimental factors (the type of herbal preparation, the type of compound, and the digestion stage), the data were submitted to an analysis of variance (ANOVA) followed by a post hoc Tukey test. The dependent samples t-test was used to compare the means for the phytochemical contents or antioxidant activity to indicate the significant differences between the PPM and DE_PPMeq_. A level of significance of *p* ≤ 0.05 was used.

## 5. Conclusions

In conclusion, our study showed that the extractability and stability of *A. uva-ursi* phytochemicals during dry extract preparation were affected by the type of the analyzed compound and the ethanol concentration applied during the extraction. Besides the basic phytochemical analyses, the bioaccessibility was determined after a simulated digestion process to better predict the pro-health potential of the raw herbal material and the dry extracts. It was revealed that the in vitro bioaccessibility of arbutin, the main bioactive compound of *A. uva-ursi*, was relatively high and unaffected by the experimental factors in the vast majority of cases. In the case of the other compounds, which can also contribute to the biological effects of bearberry products, the bioaccessibility was more differentiated and depended on the stage of digestion (gastric or intestinal), the type of herbal preparation subjected to digestion (powdered plant material or dry extracts), and the dry extract variant. The present results suggest the possibility of using dry extracts prepared with 40% ethanol for phytopharmaceutical or functional food supplement purposes as a compromise between the abundance, antioxidant activity, and potential bioaccessibility of the main phytochemicals. However, given the potential multitude of host-related factors that may occur in a living organism, in vivo studies on the bioavailability and bioactivity are needed to confirm the effects observed in vitro and to establish the biological significance of *A. uva-ursi* herbal products, especially considering the modification of their phytochemical profile via preparation techniques.

## Figures and Tables

**Table 1:** Characterization of the herbal preparations—content of individual phytochemicals and the stability during the extract drying.

	Extract	PPM	DE	DE_PPMeq_	Stability (%)
Arbutin (mg/g)	W	86.69 ± 1.05 ^a^*	199.11 ± 1.24 ^a^	81.53 ± 0.51 ^a^^	94.0 ± 1.72 ^AB^
	20E	86.18 ± 0.73 ^ab^	160.52 ± 2.66 ^b^	82.51 ± 1.37 ^a^^	95.7 ± 2.40 ^AB^
	40E	84.91 ± 0.52 ^ab^	152.53 ± 4.97 ^bc^	81.08 ± 2.64 ^ab^^	95.5 ± 3.70 ^A^
	60E	84.82 ± 0.66 ^ab^	147.22 ± 2.02 ^c^	81.86 ± 1.13 ^a^^	96.5 ± 2.08 ^A^
	80E	84.25 ± 0.79 ^b^	146.53 ± 4.26 ^c^	82.10 ± 2.39 ^a^^	97.5 ± 3.74 ^A^
	E	76.27 ± 0.44 ^c^	154.81 ± 6.61 ^bc^	75.85 ± 3.24 ^b^^	99.4 ± 4.82 ^A^
Hydroquinone (mg/g)	W	14.40 ± 0.26 ^b^	27.35 ± 1.13 ^a^	11.20 ± 0.46 ^b^^	77.8 ± 4.64 ^bC^
	20E	18.17 ± 0.34 ^a^*	29.58 ± 1.82 ^a^	15.20 ± 0.93 ^a^^	83.7 ± 6.69 ^bB^
	40E	10.99 ± 0.15 ^c^	19.80 ± 0.73 ^b^	10.53 ± 0.39 ^b^^	95.8 ± 4.80 ^abA^
	60E	7.09 ± 0.11 ^d^	12.54 ± 0.53 ^c^	6.97 ± 0.29 ^c^^	98.3 ± 5.68 ^abA^
	80E	5.28 ± 0.15 ^e^	9.33 ± 0.21 ^d^	5.22 ± 0.12 ^d^^	99.0 ± 5.07 ^aA^
	E	3.71 ± 0.12 ^f^	7.46 ± 0.15 ^e^	3.65 ± 0.07 ^e^^	98.4 ± 5.12 ^aAB^
Hyperoside (mg/g)	W	9.74 ± 0.16 ^b^	22.71 ± 0.24 ^a^	9.30 ± 0.10 ^bc^^	95.4 ± 2.53 ^A^
	20E	10.37 ± 0.11 ^a^*	20.15 ± 0.97 ^b^	10.36 ± 0.50 ^a^^	99.9 ± 5.86 ^A^
	40E	10.35 ± 0.18 ^a^	19.42 ± 0.64 ^b^	10.33 ± 0.34 ^a^^	99.8 ± 5.04 ^A^
	60E	9.98 ± 0.15 ^ab^	17.81 ± 0.63 ^c^	9.90 ± 0.35 ^ab^^	99.2 ± 4.98 ^A^
	80E	9.86 ± 0.14 ^b^	17.42 ± 0.48 ^c^	9.76 ± 0.27 ^ab^^	99.0 ± 4.12 ^A^
	E	8.78 ± 0.13 ^c^	17.79 ± 0.19 ^c^	8.72 ± 0.09 ^c^^	99.3 ± 2.52 ^A^
Pentagalloylglucose (mg/g)	W	3.31 ± 0.04 ^c^	6.96 ± 0.36 ^c^	2.85 ± 0.15 ^d^^	86.0 ± 5.53 ^ABC^
	20E	4.63 ± 0.05 ^b^	8.74 ± 0.24 ^b^	4.49 ± 0.12 ^c^^	97.0 ± 3.74 ^A^
	40E	6.59 ± 0.13 ^a^*	12.02 ± 0.14 ^a^	6.39 ± 0.08 ^a^^	96.9 ± 3.06 ^A^
	60E	6.55 ± 0.24 ^a^	11.47 ± 0.39 ^a^	6.38 ± 0.22 ^a^^	97.3 ± 6.87 ^A^
	80E	6.54 ± 0.08 ^a^	11.23 ± 0.36 ^a^	6.29 ± 0.20 ^a^^	96.2 ± 4.16 ^A^
	E	6.24 ± 0.14 ^a^	11.66 ± 0.28 ^a^	5.71 ± 0.14 ^b^^	91.6 ± 4.26 ^AB^
Methylarbutin (mg/g)	W	2.45 ± 0.04 ^c^	5.32 ± 0.14 ^b^	2.18 ± 0.06 ^c^^	89.1 ± 3.92 ^AB^
	20E	3.68 ± 0.08 ^a^*	6.34 ± 0.15 ^a^	3.26 ± 0.08 ^a^^	88.6 ± 4.04 ^AB^
	40E	2.73 ± 0.07 ^b^	4.81 ± 0.09 ^c^	2.55 ± 0.05 ^b^^	93.6 ± 4.05 ^A^
	60E	2.71 ± 0.07 ^b^	4.54 ± 0.17 ^c^	2.53 ± 0.10 ^b^^	93.3 ± 5.95 ^A^
	80E	2.73 ± 0.08 ^b^	4.63 ± 0.08 ^c^	2.59 ± 0.04 ^b^^	95.1 ± 4.54 ^A^
	E	2.70 ± 0.08 ^b^	5.27 ± 0.20 ^b^	2.58 ± 0.10 ^b^^	95.9 ± 6.52 ^AB^
Picein (mg/g)	W	2.04 ± 0.03 ^a^*	4.20 ± 0.06 ^a^	1.72 ± 0.02 ^a^^	84.3 ± 2.48 ^bBC^
	20E	1.79 ± 0.03 ^b^	3.34 ± 0.09 ^b^	1.72 ± 0.05 ^a^^	96.2 ± 4.53 ^abAB^
	40E	1.84 ± 0.05 ^b^	3.33 ± 0.10 ^b^	1.77 ± 0.05 ^a^^	96.2 ± 5.46 ^abA^
	60E	1.80 ± 0.07 ^b^	3.19 ± 0.12 ^bc^	1.77 ± 0.07 ^a^^	98.3 ± 7.27 ^aA^
	80E	1.79 ± 0.04 ^b^	3.17 ± 0.05 ^bc^	1.78 ± 0.03 ^a^^	99.0 ± 3.97 ^aA^
	E	1.74 ± 0.03 ^b^	3.04 ± 0.10 ^c^	1.49 ± 0.05 ^b^^	85.5 ± 4.45 ^bB^

Data represent the means (*n* = 3) ± SD. Means followed by different superscript lowercase letters in the columns for the PPM, DE, and DE_PPMeq_, and the stability within the same compound, differ significantly. The caret (^^^) for DE_PPMeq_ indicates a statistically significant difference from the PPM for the same extractant variant. Different superscript uppercase letters for stability indicate statistically significant differences between compounds within the same type of extractant. W—water extract, 20E—20% ethanol extract, 40E—40% ethanol extract, 60E—60% ethanol extract, 80E—80% ethanol extract, and E—100% ethanol extract; PPM—powdered plant material, DE—dry extract, and DE_PPMeq_—powdered plant material equivalent for the dry extract. * Regarding the different extractabilities of the compounds affected by the applied solvent variant, the indicated values represent the assumed abundance of individual phytochemicals in the initial powdered plant material (before digestion) for the bioaccessibility determination.

**Table 2:** Characterization of herbal preparations—the total phenolic content, antioxidant activity, and the stability of the phytochemicals during the extract drying.

	Extract	PPM	DE	DE_PPMeq_	Stability (%)
Total Phenolic Content (mg GAE/g)	W	181.15 ± 6.53 ^c^	396.49 ± 6.57 ^c^	162.34 ± 2.69 ^c^^	89.6 ± 4.72
	20E	244.14 ± 3.34 ^ab^	444.54 ± 13.21 ^b^	228.50 ± 6.79 ^b^^	93.6 ± 4.06
	40E	256.79 ± 7.40 ^a^	480.12 ± 15.01 ^a^	255.23 ± 7.98 ^a^^	99.4 ± 5.98
	60E	261.31 ± 6.32 ^a^*	467.19 ± 11.24 ^ab^	259.78 ± 6.25 ^a^^	99.4 ± 4.80
	80E	257.70 ± 7.36 ^a^	454.74 ± 10.77 ^ab^	254.79 ± 6.03 ^a^^	98.9 ± 5.17
	E	231.73 ± 7.73 ^b^	451.98 ± 6.89 ^ab^	221.43 ± 3.38 ^b^^	95.6 ± 4.65
ABTS^•+^ Scavenging Activity (mg TE/g)	W	639.32 ± 14.45 ^c^	1464.95 ± 33.53 ^ab^	599.82 ± 13.73 ^d^^	93.8 ± 4.27
	20E	801.19 ± 21.12 ^ab^	1466.22 ± 39.92 ^ab^	753.66 ± 20.52 ^b^^	94.1 ± 5.04
	40E	834.97 ± 11.22 ^a^*	1544.36 ± 26.26 ^a^	820.97 ± 13.96 ^a^^	98.3 ± 2.99
	60E	823.62 ± 20.73 ^a^	1456.29 ± 33.76 ^b^	809.78 ± 18.77 ^a^^	98.3 ± 4.76
	80E	822.98 ± 18.85 ^a^	1444.03 ± 18.44 ^b^	809.08 ± 10.33 ^a^^	98.3 ± 3.51
	E	761.14 ± 23.85 ^b^	1488.08 ± 20.77 ^ab^	729.04 ± 10.17 ^b^^	95.8 ± 4.34
Fe^3+^-Reducing Power (mg TE/g)	W	239.82 ± 6.14 ^c^	200.10 ± 5.59 ^d^	200.10 ± 5.59 ^d^^	83.4 ± 4.47
	20E	320.55 ± 9.50 ^a^	310.98 ± 9.45 ^a^	310.98 ± 9.45 ^a^^	97.0 ± 5.83
	40E	326.15 ± 10.26 ^a^*	318.56 ± 9.87 ^a^	318.56 ± 9.87 ^a^^	97.7 ± 6.11
	60E	313.74 ± 12.21 ^a^	298.98 ± 6.09 ^ab^	298.98 ± 6.09 ^ab^^	95.3 ± 5.66
	80E	303.83 ± 12.04 ^a^	281.93 ± 13.63 ^b^	281.93 ± 13.63 ^b^^	92.8 ± 8.18
	E	270.18 ± 12.01 ^b^	243.23 ± 11.41 ^c^	243.23 ± 11.41 ^c^^	90.0 ± 8.24

Data represent the means (*n* = 3) ± SD. Means followed by different superscript lowercase letters in the columns for the PPM, DE, and DE_PPMeq_ within the same assay differ significantly. The caret (^^^) for DE_PPMeq_ indicates a statistically significant difference from the PPM for the same extractant variant. W—water extract, 20E—20% ethanol extract, 40E—40% ethanol extract, 60E—60% ethanol extract, 80E—80% ethanol extract, and E—100% ethanol extract; PPM—powdered plant material, DE—dry extract, and DE_PPMeq_—powdered plant material equivalent for dry extract. * Regarding the different extractabilities of the compounds affected by the applied solvent variant, the indicated values represent the assumed abundance of individual phytochemicals in the initial powdered plant material (before digestion) for the bioaccessibility determination.

## Data Availability

The data presented in this study are available on request from the corresponding author.

## Supplementary Materials

The following supporting information can be downloaded at: https://www.mdpi.com/article/10.3390/molecules29245968/s1, Table S1: Dry extract yield and plant material/extract ratio; Table S2: Standard curve parameters for the HPLC analysis of bearberry phytochemicals; Figure S1: HPLC-UV chromatogram of standard compound mixture; Figures S2–S7: HPLC-UV chromatograms of bearberry phytochemicals.
