# Supplementary material for: Effect of the Type of Herbal Preparations (Powdered Plant Material vs. Dry Ethanolic Extracts) on the Bioaccessibility of Bearberry (Arctostaphylos uva-ursi (L.) Spreng.) Phytochemicals in Simulated Digestion Conditions
Source: Molecules. 2024 Dec 18;29(24):5968. doi: 10.3390/molecules29245968 (PMC11678513; doi:10.3390/molecules29245968)
Supplement: Supplementary file 1 [file molecules-29-05968-s001.zip › molecules-3330612-supplementary.pdf]

Table S1. Dry extract yield and plant material/extract ratio.

Type of dry extract	Dry extract yield (g DE/g PPM)	Plant material/extract ratio (g PPM/g DE)
W	0.409±0.0006[f]	2.442±0.0035[a]
20E	0.514±0.0009[d]	1.945±0.0036[c]
40E	0.532±0.0009[c]	1.881±0.0031[d]
60E	0.556±0.0010[b]	1.798±0.0033[e]
80E	0.560±0.0009[a]	1.785±0.0029[f]
E	0.490±0.0009[e]	2.041±0.0039[b]

Data represent means ± SD. Means followed by different superscript lowercase letters in the columns differ significantly. W – water extract, 20E – 20% ethanol extract, 40E – 40% ethanol extract, 60E – 60% ethanol extract, 80E – 80% ethanol extract, E – 100% ethanol extract; DE – dry extract, PPM – powdered plant material.

Table S2. Standard curve parameters for the HPLC analysis of bearberry phytochemicals.

Compound	Tested range (μg/mL)	Regression equation	R^2	LOD (μg/mL)	LOQ (μg/mL)
Arbutin	1.5625-200	y = 0.1421x + 0.1447	0.99998	1.150	3.484
Hydroquinone	1.5625-200	y = 0.3570x + 0.5011	0.99996	1.504	4.556
Picein	1.5625-100	y = 0.7811x + 0.9613	0.99995	0.925	2.804
Methylarbutin	1.5625-100	y = 0.1345x + 0.0073	0.99996	0.792	2.399
Hyperoside	1.5625-100	y = 0.8010x + 0.7028	0.99993	1.079	3.270
Pentagalloylglucose	1.5625-100	y = 1.4537x + 0.6011	0.99994	1.023	3.099

Limit of detection (LOD) and limit of quantification (LOQ) were calculated as 3.3σ/S and 10σ /S, respectively.

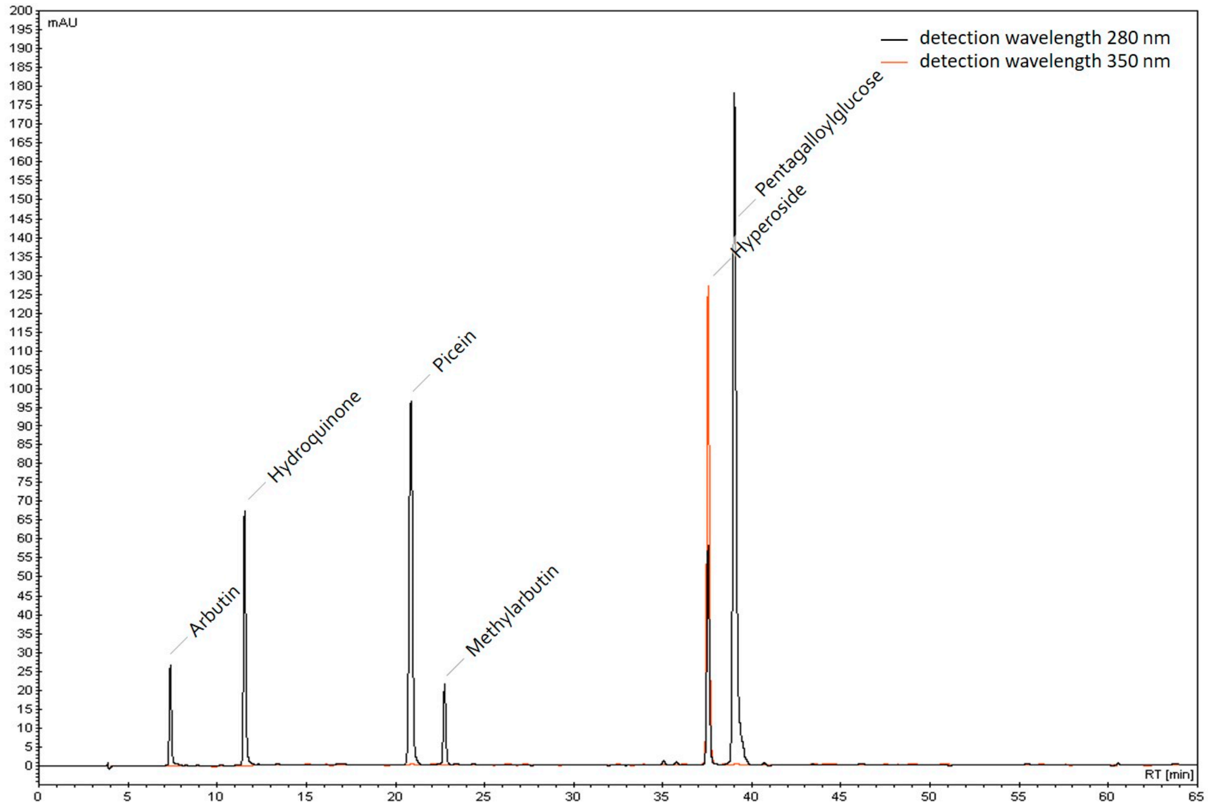

Figure S1. HPLC-UV chromatogram of standard compound mixture.
Data were collected at 280 nm (black line) for arbutin, hydroquinone, pentagalloylglucose, methylarbutin, and picein and at 350 nm (orange line) for hyperoside. mAU – milli-absorbance unit; RT – retention time.

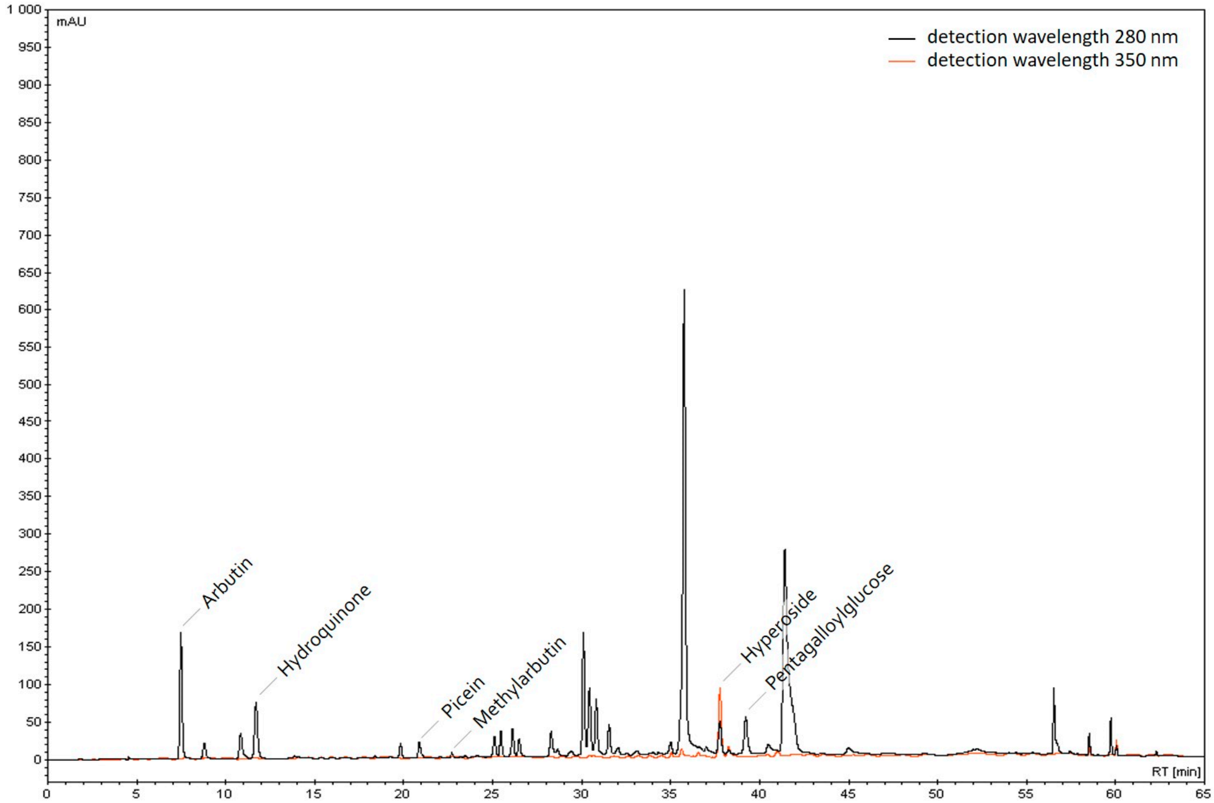

Figure S2. HPLC-UV chromatogram of bearberry phytochemicals from water extract.
Data were collected at 280 nm (black line) for arbutin, hydroquinone, pentagalloylglucose, methylarbutin, and picein and at 350 nm (orange line) for hyperoside. mAU – milli-absorbance unit; RT – retention time.

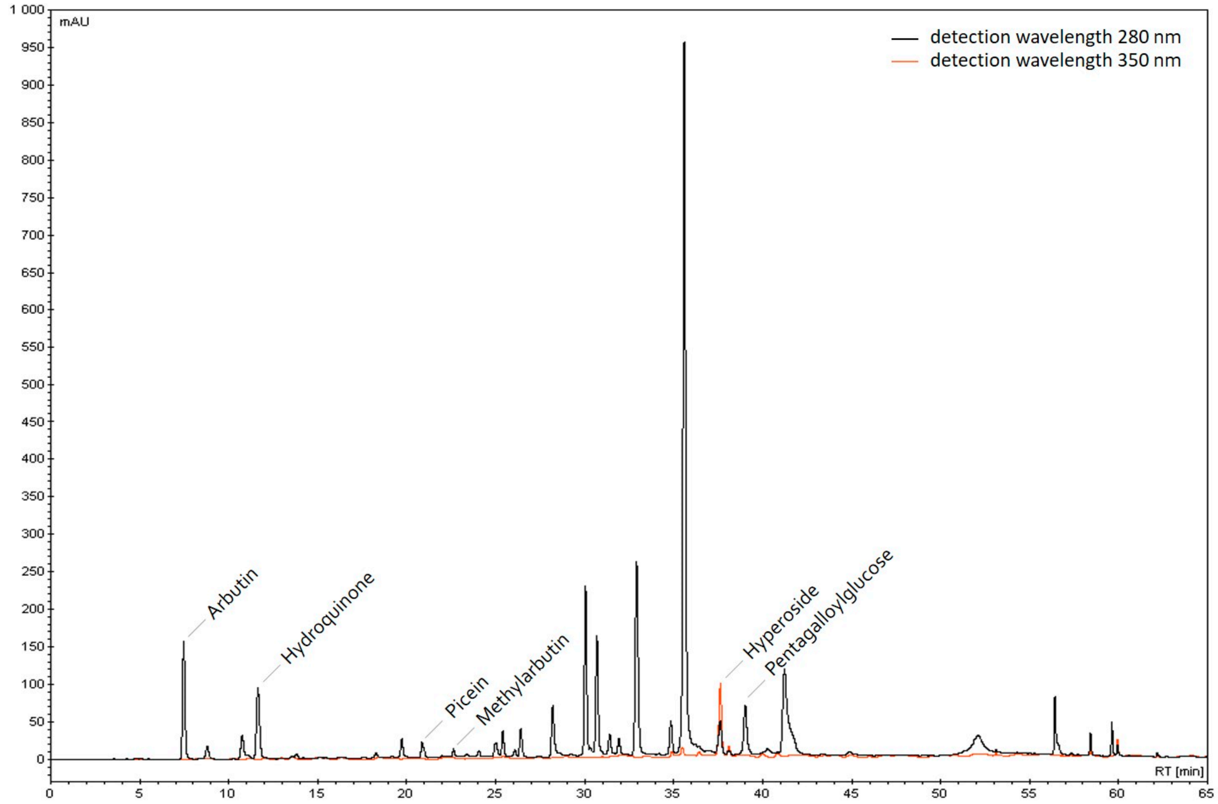

Figure S3. HPLC-UV chromatogram of bearberry phytochemicals from 20% ethanol extract.
Data were collected at 280 nm (black line) for arbutin, hydroquinone, pentagalloylglucose, methylarbutin, and picein and at 350 nm (orange line) for hyperoside. mAU – milli-absorbance unit; RT – retention time.

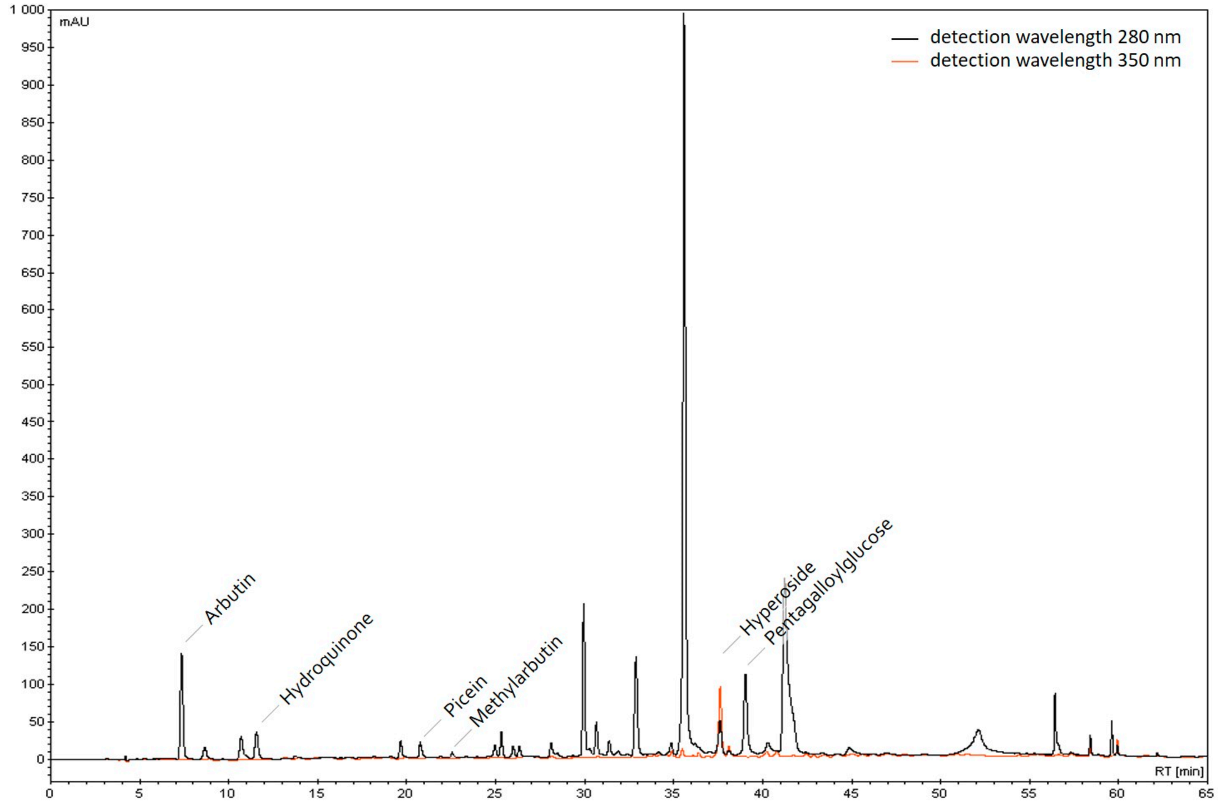

Figure S4. HPLC-UV chromatogram of bearberry phytochemicals from 40% ethanol extract.
Data were collected at 280 nm (black line) for arbutin, hydroquinone, pentagalloylglucose, methylarbutin, and picein and at 350 nm (orange line) for hyperoside. mAU – milli-absorbance unit; RT – retention time.

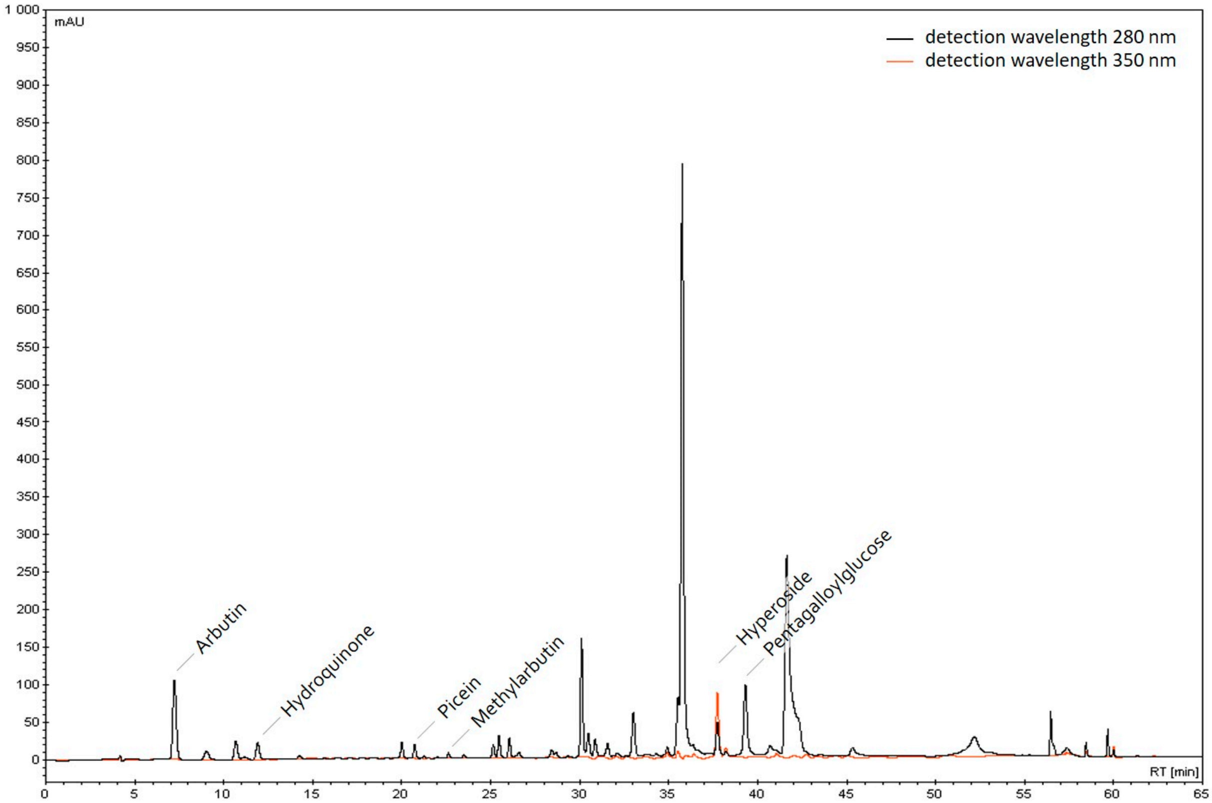

Figure S5. HPLC-UV chromatogram of bearberry phytochemicals from 60% ethanol extract.
Data were collected at 280 nm (black line) for arbutin, hydroquinone, pentagalloylglucose, methylarbutin, and picein and at 350 nm (orange line) for hyperoside. mAU – milli-absorbance unit; RT – retention time.

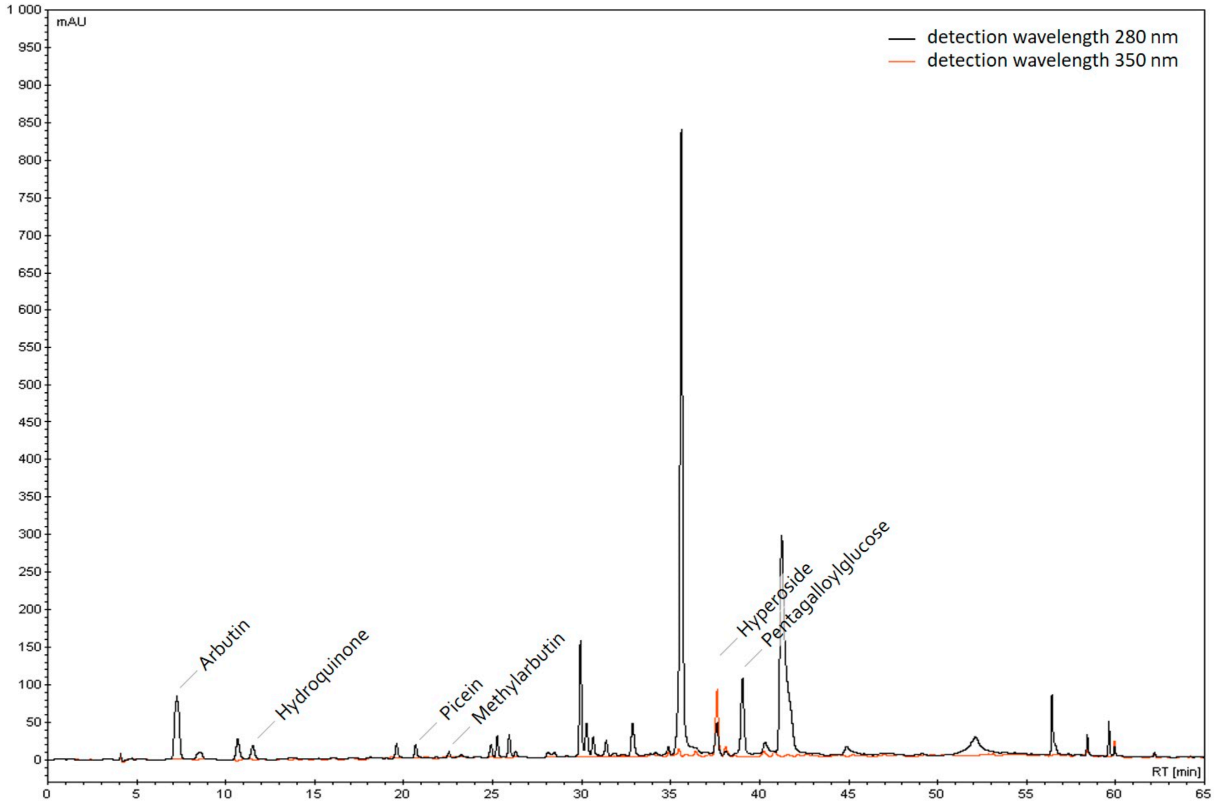

Figure S6. HPLC-UV chromatogram of bearberry phytochemicals from 80% ethanol extract.
Data were collected at 280 nm (black line) for arbutin, hydroquinone, pentagalloylglucose, methylarbutin and picein, and at 350 nm (orange line) for hyperoside. mAU – milli-absorbance unit; RT – retention time.

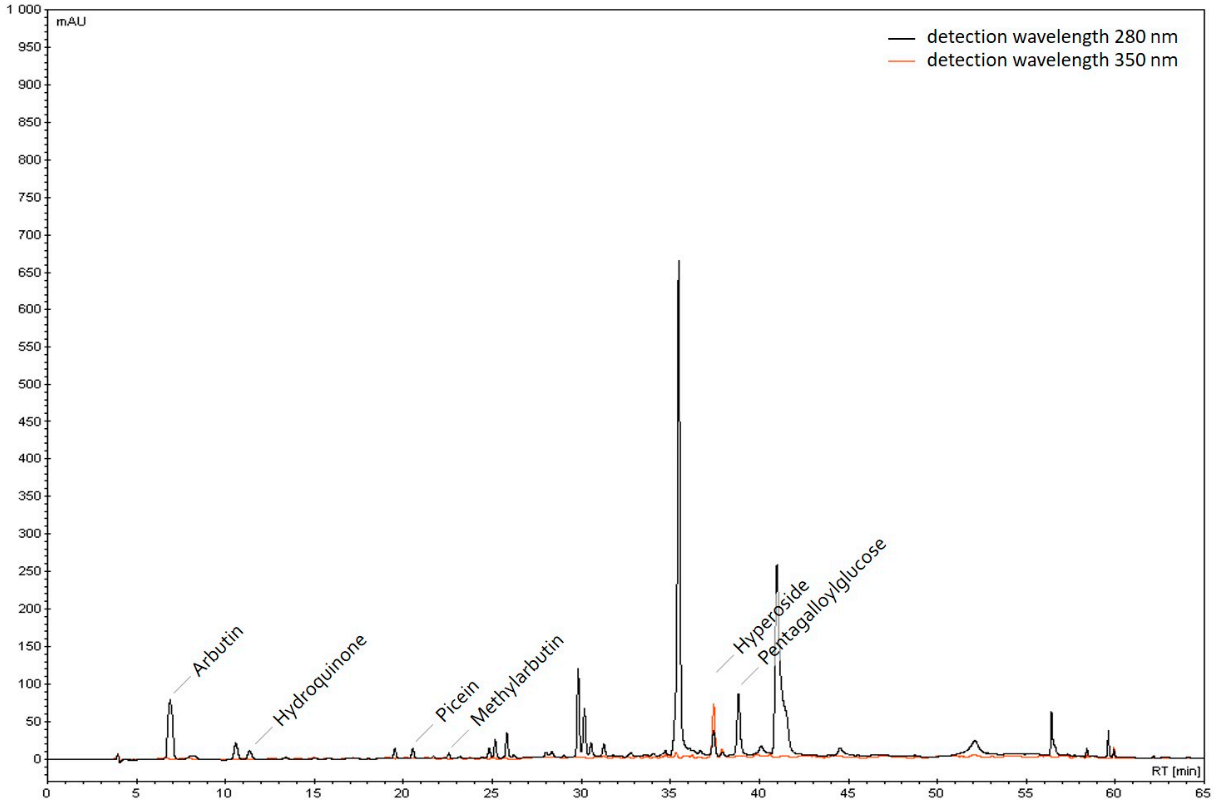

Figure S7. HPLC-UV chromatogram of bearberry phytochemicals from ethanol extract.
Data were collected at 280 nm (black line) for arbutin, hydroquinone, pentagalloylglucose, methylarbutin and picein, and at 350 nm (orange line) for hyperoside. mAU – milli-absorbance unit; RT – retention time.
